# Towards a Theory of Compassion Fatigue in Palliative Care and Oncology: A Systematic Scoping Review

**DOI:** 10.1177/10499091251315183

**Published:** 2025-01-18

**Authors:** Annushkha Sinnathamby, Halah Ibrahim, Yun Ting Ong, Nila Ravindran, Darius Wei Jun Wan, Jun Hao Tan, Nur Amira Binte Abdul Hamid, Nagavalli Somasundaram, Simon Yew Kuang Ong, Lalit Kumar Radha Krishna

**Affiliations:** 1 Khoo Teck Puat National University Children’s Medical Institute, 150744 National University Health System, Singapore; 2 Division of Supportive and Palliative Care, 203378 National University Cancer Institute Singapore, Singapore; 3 Division of Supportive and Palliative Care, 68751 National Cancer Centre Singapore, Singapore; 4 Division of Cancer Education, 68751 National Cancer Centre Singapore, Singapore; 5 Department of Medical Science, 623879 Khalifa University College of Medicine and Health Sciences, Abu Dhabi, United Arab Emirates; 6 Yong Loo Lin School of Medicine, 63751 National University of Singapore, Singapore; 7 371018 Lee Kong Chian School of Medicine, Nanyang Technological University, Singapore; 8 Division of Medical Oncology, 68751 National Cancer Centre Singapore, Singapore; 9 Duke-NUS Medical School, National University of Singapore, Singapore; 10 Centre for Biomedical Ethics, National University of Singapore, Singapore; 11 Palliative Care Institute Liverpool, Academic Palliative & End of Life Care Centre, Cancer Research Centre, 4591 University of Liverpool, Liverpool, UK; 12 PalC, The Palliative Care Centre for Excellence in Research and Education, Singapore

**Keywords:** palliative care, oncology, burnout, compassion fatigue, vicarious trauma, secondary traumatic stress, costs of caring

## Abstract

**Background:**

In their care of terminally ill patients, palliative care physicians and oncologists are increasingly predisposed to physical and emotional exhaustion, or compassion fatigue (CF). Challenges faced by physicians include complex care needs; changing practice demands, and sociocultural contextual factors. Efforts to better understand CF have, however, been limited. We propose a systematic scoping review (SSR) to determine “What is known about theories of CF in physicians?”.

**Methods:**

Guided by the PRISMA-based Systematic Evidence-based Approach (SEBA) methodology, our SSR comprised searches for articles published between 1 January 2000 and 31 December 2023 on MEDLINE, EMBASE, PsycINFO, Wiley, CINAHL and Google Scholar databases. Both thematic and content analyses were carried out.

**Results:**

Of the 10 505 titles identified, 80 articles were included. 15 current theories of CF were evaluated, leading to two key domains: theories of CF and theories related to the costs of caring. Overall, theories of CF evolved from Figley’s model with gradual encompassing of moral distress, vicarious trauma and burnout, alongside the inclusion of individual characteristics, decisioning and nous in later theories.

**Conclusion:**

CF was found to be part of a wider cost of caring that links clinical experiences with self-concepts of personhood and identity. The Ring Theory of Personhood has been able to shed light on how physicians will respond to such experiences and is key to guiding physician support and the creation of nurturing working environments.

## Introduction

Journeying with terminally ill patients^1-7^ and their families leave palliative care physicians and oncologists prone to physical and emotional exhaustion.^3,6,8^ This risk of compassion fatigue (CF) is compounded by sociocultural, psychoemotional and practice influences^9-13^ and complicated by the wider roles taken by these physicians in communicating,^14^ caring and concurrently supporting patients^15-17^ and their families/caregivers, members of the multidisciplinary team^1,2,18,19^ and trainees.^8,20^ These roles predispose palliative care physicians and oncologists to vicarious trauma (VT)—distress caused by empathizing with staff, patient and/or family distress—and secondary traumatic stress^21^ (STS), described as the blurring of boundaries between personal and professional lives as a result of witnessing the suffering of patients and their families and/ or staff distress.^22,23^

Concurrently, functioning in a complex, often resource-limited and demanding environment has also seen palliative care physicians and oncologists face a more complicated form of moral distress (MD) that brings about a sense of powerlessness to act ethically — threatening a physician’s moral integrity. This wider concept of MD^18,24^ also includes the influence of the patient, family, healthcare team and physician’s own perceptions, meaning-making, psycho-emotional considerations, resilience, coping strategies and support mechanisms. This wider notion of MD is apt when considering accounts from Southeast Asia^22,23,25,26^ that suggest that physicians report MD due to their perception of being made to participate, collaborate and perpetuate practices that might run against their instincts, beliefs, values, norms and principles (henceforth belief systems). This includes practices such as collusion,^27,28^ familial determination,^29-33^ circumnavigation of direct patient involvement in care determinations and adjustments to treatment modalities^34^ and care options^34-36^ to meet familial interests.^37-42^ Whilst these practices are consistent with local culture, they run contrary to Western notions of autonomy and individualism inculcated during medical training.^32,33,43^ This may lead to conflicts in some physicians. The presence, severity and nature of the distress suffered^15,16,44^ underscore the influence of the physician’s belief systems and contextual considerations. When present, this distress can precipitate feelings of frustration, anxiety, distress and worry.^25^ Unsurprisingly, the wider notion of MD encapsulates a highly personalized notion of moral, ethical and existential distress tempered by the physician’s narrative, experiences, meaning-making, training, competency, attitudes and abilities (nous), psycho-emotional well-being,^45^ belief systems and contextual and environmental factors.

Together, the wider overlapping work-related concepts of MD and CF, along with acknowledgment of the entwined nature of their relationships with recurrent episodes of STS, VT and burnout, suggest the need to reconsider current thinking if healthcare teams are to better support a physician’s resilience, meaning-making and coping^46-67^ and safeguard the provision of compassionate and holistic patient care.^68^

We propose a systematic scoping review (SSR) to determine “What is known about theories of compassion fatigue in physicians?”. We posit that a theory of CF, or “an organized body of concepts and principles intended to explain CF”, will platform better understanding of the costs of caring. We also postulate that these new insights will scaffold the design of better assessments and supportive measures for palliative care physicians, oncologists^69^ and other members of the healthcare team.^70^

## Methods

This SSR on current theories of CF in physicians was conducted using the Systematic Evidence-Based Approach (SEBA) due to its established role in examining shifts in practice, thinking and perception.^2,3,14,71-74^ Moreover, SEBA’s constructivist approach and relativist lens support the interpretation of CF as a sociocultural construct influenced by diverse psychological, relational and situational factors, thus enabling a more holistic review that captures varied perspectives.

### Stage 1 of SEBA – Systematic Scoping Review

Guided by the PRISMA-ScR guidelines and the Population, Context and Concept (PCC) framework, the SSR began with defining the primary research question: “What is known about theories of compassion fatigue in physicians?”. The inclusion and exclusion criteria are detailed in Table 1.

**Table 1.** Population, Context and Concept (PCC) Framework and Inclusion and Exclusion Criteria Applied to Database Search.

	Inclusion criteria	Exclusion criteria
Population	Practising physicians in medical specialties	Papers that discuss medical students or residents, physicians in non-medical specialties
		Allied health specialties
		Clinical and translational science, veterinary, dentistry
Context	Clinical practice	Clinical research or academics
	All study designs: qualitative, quantitative, and mixed study methods	
	Systematic reviews, literature reviews, and narrative reviews, grey literature	
	Year: January 1, 2000–December 31, 2023	
Concept	Papers discussing CF including factors influencing CF and relationships with related concepts: STS, VT, burnout and compassion satisfaction	Papers that do not discuss CF
	Papers that discuss how to address CF and related concepts; outcomes of CF and related concepts	
	Papers that discuss existing theories of CF and related concepts	

Searches were conducted between 17th December 2023 and 17th January 2024 by two independent research teams using the search strategy featured in Table 2. Articles published between 1st January 2000 and 31st December 2023 in Embase, PsycINFO, MEDLINE, CINAHL and Google Scholar were reviewed. Frequently cited theories published before 2000 were also included through snowballing to capture foundational perspectives. Abstracts were screened using EndNote, with consensus on the final lists of articles achieved through Sandelowski and Barroso’s^75^ “negotiated consensual validation”.

**Table 2.** Example of Search Strategy Applied to PubMed and Google Scholar Database Search.

PubMed	
After publication date filter (from 1^st^ Jan 2000 to 31^st^ December 2023) and language filter (English)	3893
(Physician*[Mesh] OR Clinician*[Title/Abstract] OR physician*[Title/Abstract] OR Doctor*[Title/Abstract]) AND (“Compassion Fatigue”[Mesh] OR compassion fatigue*[Title/Abstract] OR empath* stress OR empath* fatigue OR ((stress*[Title/Abstract] OR trauma* [Title/Abstract] OR fatigue) AND (secondary[Title/Abstract] OR empath* [Title/Abstract] OR vicarious[Title/Abstract])))	
Google Scholar	
After publication date filter (from 2000 to 2023) and language filter (English)	86
allintitle: doctor OR doctors OR physician OR physicians OR residents OR resident OR clinician OR clinicians “compassion fatigue”	

### Stage 2 of SEBA – Split Approach

The Split Approach combines Braun and Clarke’s^76^ thematic analysis and Hsieh and Shannon’s^77^ directed content analysis to facilitate a robust and multi-dimensional analysis.

Following Braun and Clarke’s^76^ method, one team of researchers conducted an inductive analysis, identifying codes from surface-level meanings in the data. Complementary codes were grouped together to form larger themes. Concurrently, a second team used a deductive approach based on a priori codes from Sinclair et al’s^78^ review of CF, ensuring consistency and thorough coverage of key concepts. Classifying codes with similar meanings formed categories. To ensure key concepts of CF were retained, a third team of researches created tabulated summaries of included articles and evaluated their quality using the Medical Education Research Study Quality Instrument^79^ (MERSQI) and Consolidated Criteria for Reporting Qualitative Research^80^ (COREQ).

### Stage 3 of SEBA – Jigsaw Perspective

This stage was guided by France et al’s^81^ adaptation of Noblit et al’s^82^ meta-ethnographic approach. The research team compared identified themes and categories systematically to determine if they could be integrated into broader interpretations. Themes and categories were visualized as pieces of a jigsaw puzzle, with complementary pieces combined to reveal larger patterns.

### Stage 4 of SEBA – Funnelling Process

The final list of themes and categories was compared against tabulated article summaries to ensure alignment and validity, creating domains that framed the discussion. This verification step helped ensure the trustworthiness and robustness of the final themes and categories.

### Stage 5 of SEBA – Analysis of Evidence-Based and Non-Data Driven Literature

Concerns on bias stemming from the use of non-peer-reviewed or non-evidence-based grey literature were mitigated by comparisons with research-based peer-reviewed data that revealed similar themes and categories between both types of data sources.

### Reflexivity and Iterative Process

Reflexivity was an essential component of the SEBA process. This saw the inclusion of an expert team of clinicians, clinician-educators and a medical librarian to advise the research process and attenuate the impact of personal experiences, assumptions, biases on data interpretation. Such reflexive insights promoted transparency and enhanced the rigor and trustworthiness of the process.

Further, SEBA’s iterative process allowed continuous adaptation to emerging findings and theories. As new theories emerged, additional data points were integrated—adapting analyses to reflect the evolving dataset.

Reflexivity and iterative adjustments were recorded in real-time, enhancing the trustworthiness of the methodology and ensuring that SEBA remained responsive and reflective of emerging insights.

## Results

**Figure 1.**
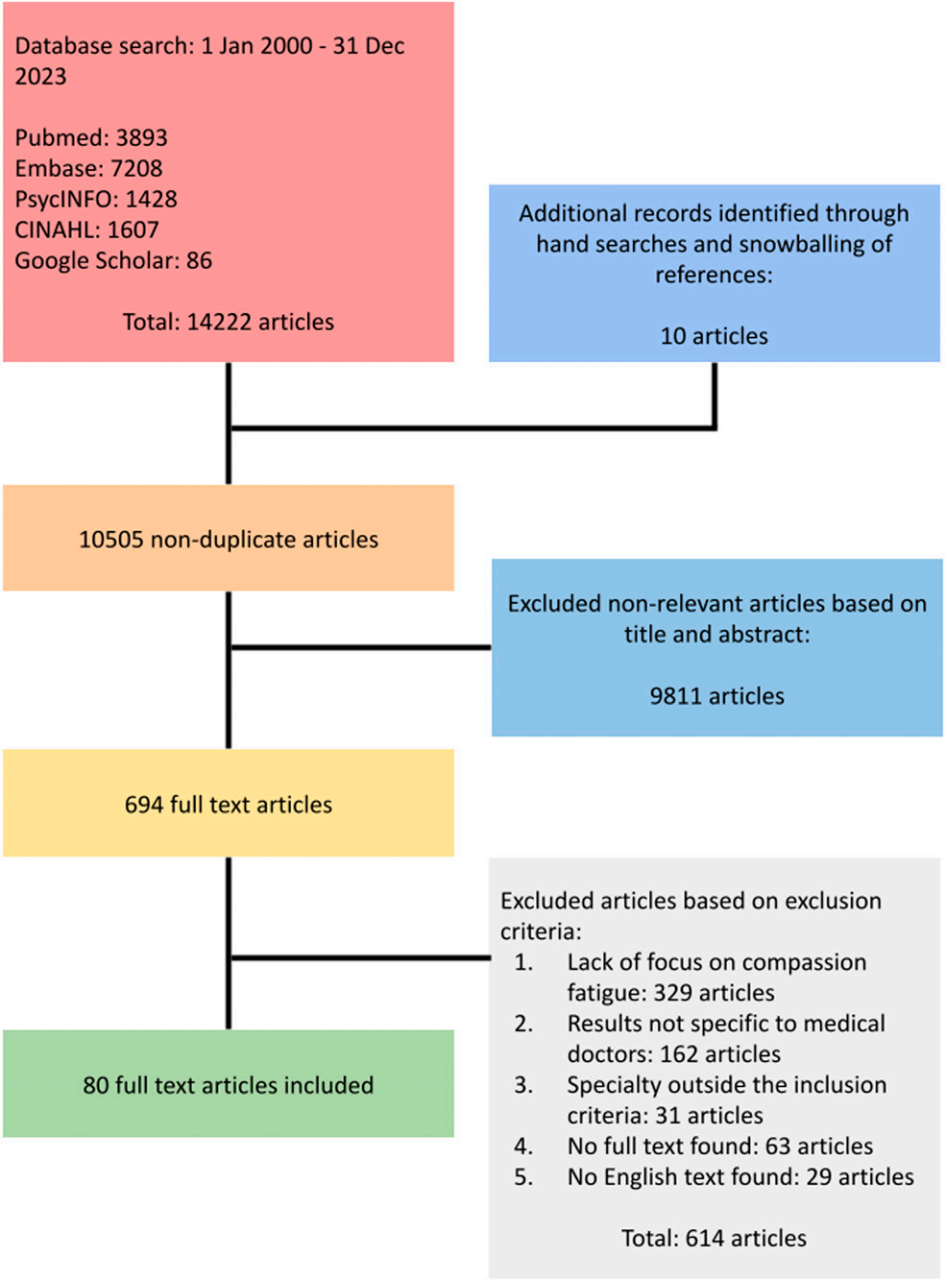
PRISMA flowchart.

Out of the 10 505 titles and abstracts identified, 694 full-text reviews were conducted, with 80 articles included in the final analysis (Figure 1). Two primary domains were identified: 1) current theories of CF, and 2) theories related to the costs of caring.

In keeping with PRISMA-ScR guidelines, our results are presented in tables for ease of reporting.

### Domain 1. Theories of Compassion Fatigue

**Figure 2.**
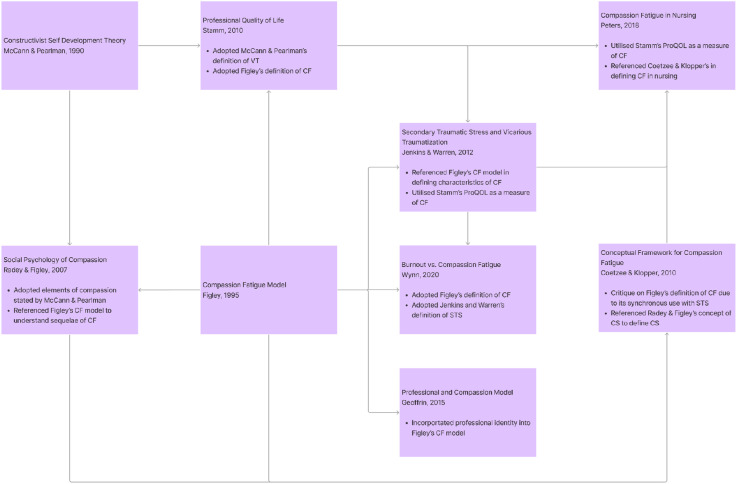
Inter-related nature of theories.

There were fifteen interpretations of pre-existing theories of CF included. Most pivot on Figley’s^68,83^ Compassion Stress and Fatigue Model and many are interrelated. This is illustrated in Figure 2.

It would thus be apt to begin with Figley’s^68,83^ Compassion Stress and Fatigue Model. This linear cause-and-effect model sees CF as a personalized experience shaped by the physician’s empathetic connections, job satisfaction, role, patient exposure, contextual and narrative history, as well as the effects of previous exposure to the suffering of others. More recent interpretations of the theory also bring to the fore the physician’s meaning-making, resilience and coping strategies, alongside environmental and contextual factors.^52,56,67,84-97^

Coetzee and Klopper’s^98^ Conceptual Framework for Compassion Fatigue takes a similar individualized and longitudinal perspective on the cumulative effects of prolonged, continuous and intense contact with patients and sources of stress. It also highlights that CF may be attenuated with effective physical, emotional and existential care and support. Synonymous with STS, Coetzee and Klopper’s^98^ notion of CF occurs when the resilience and protective factors are overwhelmed. It begins with temporary *compassion discomfort* which is reversible with rest and tapping into protective factors or supportive networks. If unabated, or if recovery has been incomplete, *compassion discomfort* creates *compassion stress. Compassion stress* brings with it poor engagement, irritability, poor output and performance and increased physical ailments.

Moving away from theories rooted in hospital care, McCann and Pearlman’s^99^ Constructivist Self Development Theory, built on the experiences of therapists working with traumatized college students, focuses on CF as a balance of dependency/trust and independence; a sense of ‘safety’ and context (or a frame of reference); and power, intimacy and esteem. Notionally, McCann and Pearlman^99^ believed that therapists constructed and internalized images of their patient’s traumatic events and experiences, precipitating VT. Scaffolded by empathy and the cumulative effect of VT is CF.

Singer and Klemicki’s^100^ theory of empathetic distress is similarly rooted in empathetic connections between physician, patient and family. Mediated by an altruistic desire^101^ to provide empathetic care, Singer and Klemicki’s^100^ theory suggests empathetic response to suffering is compassion for others, or empathic distress, which refers to “a strong aversive and self-oriented response to the suffering of others, accompanied by the desire to withdraw from a situation in order to protect oneself from excessive negative feelings”. With empathetic distress motivated to reduce negative symptoms that follow VT, the theory suggests that curbing the amount of empathy may protect from CF.

Stamm’s^102,103^ Professional Quality of Life Model suggests that therapists suffer CF as a result of an imbalance of positive (compassion satisfaction) and negative (compassion fatigue) experiences and meaning. Like STS and with a potential to precipitate burnout, newer interpretations of CF draw on how an individual makes meaning of their experiences. Positive experiences, nature of the therapeutic relationship and presence of effective professional and personal support networks that guide meaning-making and shepherd emotional effects reinforce compassion satisfaction. This intertwined relationship between CF and burnout is echoed in Wynn’s^104^ concept analysis of burnout vs CF.

Wu’s^105^ Second Victim Theory forwarded in a letter briefly suggests that traumatic interactions and errors leave physicians feeling guilty, shamed, less confident and anxious. This leads to compromises in care if inadequately addressed. While this concept does not discuss CF, it alludes to cumulative effects and the influence of poor support and an ‘unsafe’ judgmental environment.

Tedeschi and Calhoun’s^106^ Post-Traumatic Growth Inventory does not discuss CF directly. However, in their design of an assessment tool to evaluate singular traumatic experience, such as rape and bereavement, and multiple traumas, such as war and cancer diagnoses, these authors advance the theory that post-traumatic development extends along self-perception, interpersonal relationships and philosophy of life.

Fernando III and Consedine’s^107^ Transactional Model of Physician Compassion similarly brings to the fore the dynamic influences of patients and their families, clinical situation, clinical context, physical environment and institutional and physician-related factors to CF and burnout. Inspired by Engel’s^108^ biopsychosocial model, Fernando III and Consedine’s^107^ Transactional Model considers the physician’s physical-psycho-emotional state, personality, past medical experience, skills, support systems, coping abilities, personal history, individualized appraisal of the clinical situation, sensitivity to suffering, ability, motivation, resources and narratives. The patient and their family’s level of engagement, motivations, expectations and conduct also impact the relationship, as does the complexity of clinical considerations, co-morbidities, adverse effects and clinical uncertainty. Similarly, the availability of resources, work demands and environment impact considerations.

Radey and Figley’s^109^ Social Psychology of Compassion builds on Figley’s^83^ notion that CF is a factor of “poor self-care, previous unresolved trauma, inability or refusal to control work stressors, and a lack of satisfaction for the work”. This theory is focused on the reciprocal relationship between affect, resources, self-care, personal discernment and judgment and their role in attenuating “negative consequences in a broader context of positive social work”.^109^ Radey and Figley^109^ promote the notion that increased compassion satisfaction boosts affect, self-care, resources and finding positive meaning in work which tip the balance in favor of reducing CF. Valent’s^110^ framework for STS similarly explores the role of coping strategies in CF whilst Jenkin and Warren’s^111^ and Peters’^112^ concept analyses of CF in nursing highlight the threat of prolonged, repetitive and high stress exposure, alongside the protective nature of psycho-emotional and spiritual health, the sanctity of the work-life boundaries and self-care.

Geoffrion’s^113^ Professional and Compassion Model similarly builds on Figley’s^83^ Compassion Fatigue Model to suggest that CF is a blend of VT, STS, accountability stress and primary trauma. Critically, this model considers the impact of CF on professional identity and the role of gender, age, exposure to violence and organizational factors (ie, violence prevention training, support from colleagues and supervisors, ‘zero tolerance’ management policy and safe physical environment) in this complex dynamic. Adding to this complexity is the physician’s level of compassion satisfaction, their accountability for risk management and the meaning made from personal and professional experiences and the distress of others (felt accountability). This underscores the uniquely individualized perspective of CF.

#### Predisposing Factors

Eight of the included papers ascribed CF to a range of antecedent factors, including prolonged work stress, chronic exposure to suffering of others, lack of self-care measures, inability to maintain professional boundaries, high stress exposure, high occupational use of self and compassion.^112^ Other predisposing factors include physical and mental fatigue, workplace imbalances and workplace attrition. Wynn^104^ argues that burnout itself is also a predisposing factor for CF whilst Coetzee and Klopper’s^98^ model suggests that CF is the product of prolonged, continuous and intense contact with patients, use of self and exposure to stress that tips the balance towards compassion stress and spiraling physical, emotional, social, spiritual and intellectual changes.

### Domain 2. Theories Related to the Costs of Caring

The theories featured thus far show a gradual embrace of CF as a coadunation of MD, CF, VT, STS and burnout. This shift is aided by acknowledgment that prolonged, intensive and multipronged psychological and environmental factors impact the physician’s belief systems—changing the physician’s identity and reshaping self-concepts of personhood, or ‘what makes you, you’. Together with more inclusive and clinically relevant concepts of MD and CF, these factors have culminated in the theory of the costs of caring.

Yet, the true extent of the costs of caring continues to develop. The recent acknowledgment that the costs of caring impact a physician’s identity and specifically their professional identity demands careful study. Indeed, these findings have massive ramifications, given that they confirm longstanding beliefs that the cumulative emotional, existential, psychological and individual effects of the costs of caring impact patient care, family experiences, interprofessional working and the physician’s wellbeing.

**Figure 3.**
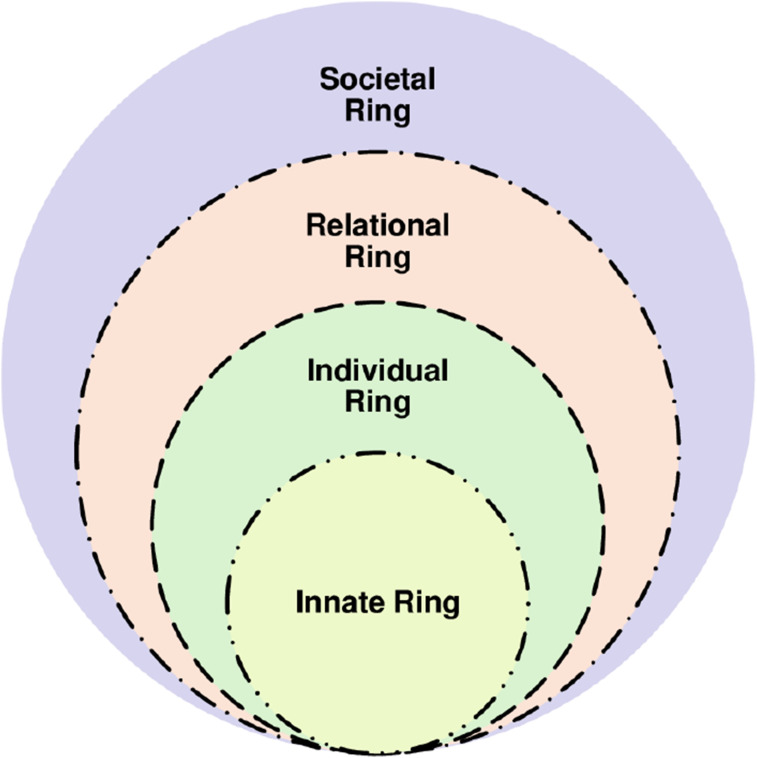
Ring theory of personhood.

To uncover the true extent of costs of caring, Somasundaram et al^69^ employed the Ring Theory of Personhood (RToP) (Figure 3).

Somasundaram et al^69^ were inspired by the posit that self-concepts of personhood depicted by the RToP’s Innate, Individual, Relational and Societal Rings, manifest in the physician’s identity and belief systems. These belief systems are a reflection of self-identity and personhood and may be discerned from their thinking, actions, conduct and practice. Each ring of the RToP captures specific aspects of the physician’s overall belief systems, described in Table 3.

**Table 3.** Description of the Ring Theory of Personhood.

Ring of Personhood	Description
Innate	Contains belief systems centered on narratives created from the physician’s spiritual/religious beliefs and demographic features, such as race and gender
Individual	Contains belief systems that guide conscious function and personality
Relational	Contains belief systems focused on close personal relationships, such as family and friends
Societal	Contains belief systems revolving around sociocultural, professional, legal and ethical norms, expectations, rights, roles and responsibilities

In addition, the RToP also recognizes the influence of the physician’s narratives, context, experience, competence, knowledge, skills, attitudes, psycho-emotional and physical states; regnant expectations; relationship with patients, family and other professionals; working environment; and practice setting in shaping the physician’s belief systems and current context-specific identity. Encapsulated within this theory is also the role played by sense-making of experiences, reflections, insights and developing nous.

These considerations make the theory of the costs of caring dynamic—underscored by its due consideration of new experiences, insights, belief systems and meaning-making on Innate, Individual, Relational and Societal belief systems, in addition to the facets discussed thus far. Through the lens of the RToP, it is possible to appreciate how the new belief systems conflict with parts of one or more belief systems. It also captures how these ‘events’ inspire adaptations to the belief systems to ensure that the identity held is contextually appropriate and in line with regnant social, ethical, legal and cultural expectations, mores and standards. Adaptations to ‘events’ must also be consistent with the physician’s core beliefs and narratives. However, when dissonance cannot be easily addressed and adaptations to belief systems are inadequate to combine the new with the regnant belief systems, there is distress. This distress may see MD complicated by emotional, existential and/or psychological distress accompanying CF, VT, STS and/or burnout. If persistent and with the absence of appropriate mentoring support, supervision and guided reflections, the effects of costs of caring may be aggravated. This underscores the cumulative effects of the costs of caring.

Even as we recognize the role of timely, personalized, appropriate and holistic help in supporting identity work, efforts to address the costs of caring also proffers the notion of a spectrum of belief systems held within the four domains. At one end of this spectrum are relatively unchanging or ‘rooted’ beliefs. At the other end, evolving belief systems that are adaptable. The adaptable aspect usually encompasses new concepts that have not yet become rooted within the physician’s self-concept of identity and personhood. The presence of evolving adaptable belief systems proffers the possibility of maladaptive belief systems that may take root without close review of individual physicians. These maladaptive belief systems would shape how physicians think, feel and act, or their professional identity formation (PIF). This not only links the costs of caring with PIF but also underlines the need for regular support of palliative care physicians and oncologists.^69^ It also underscores the need for structuring and longitudinal support of these physicians. To this end, the authors suggest the possibility of a RToP-based tool to direct support of these physicians and to inform host organizations of the need for nurturing working environments.

## Discussion

**Figure 4.**
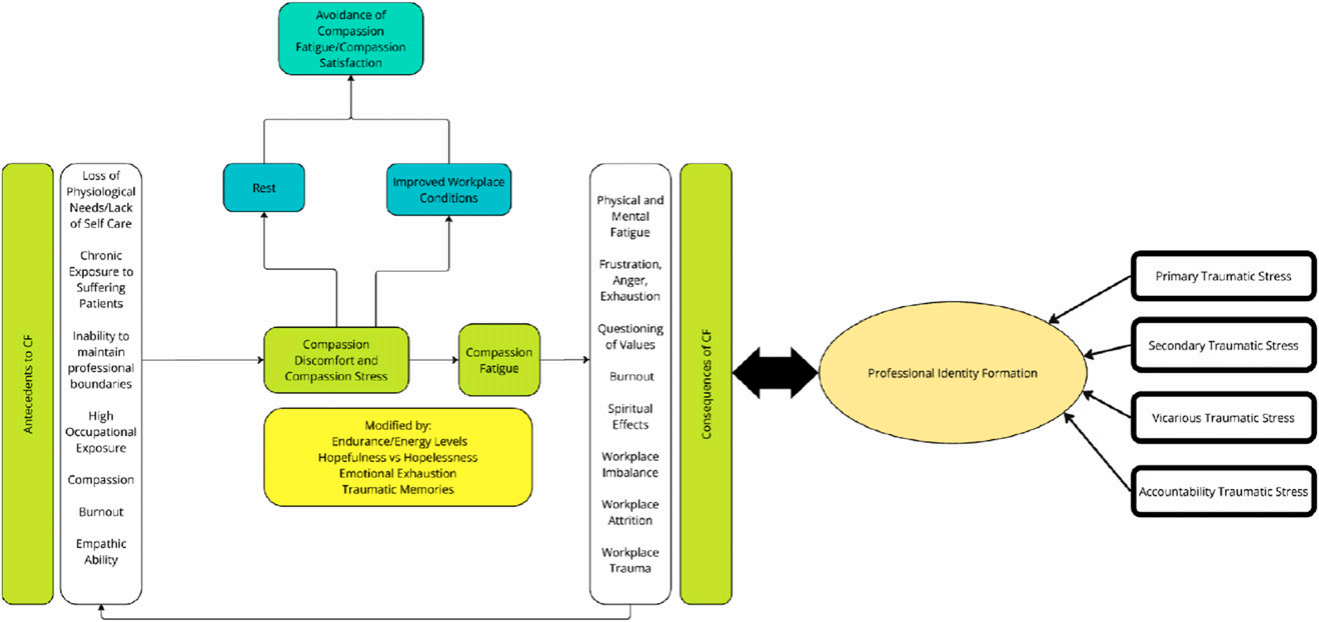
Summary of regnant theories.

Whilst the theory of the costs of caring appears to be an evolution of regnant theories of CF, there is still much to be drawn from prevailing concepts and evolutions of Figley’s^68^ Compassion Stress and Fatigue model. Perhaps most significantly, these models have begun to recognize the limitations in the concept that they seek to explain. A wider concept has been in the offing, with CF, VT, STS and burnout increasingly conflated, alongside recognition of the influence of environmental, relational and individual features and acknowledgment that the intensity, frequency, context, and sociocultural considerations play significant roles in the development of CF. The interrelatedness of the theories highlighted in Figure 4 below also platforms the proffering of the costs of caring.

Whilst the costs of caring underscore the need for regular, longitudinal and personalized support, the RToP presents a means of assessing these changes. However, perhaps a more significant contribution made by the RToP is better appreciation of what new belief systems are introduced and integrated into prevailing concepts of self and how they impact the physician’s practice and professional development, and identity. This link allows a better understanding of not only how and why, but perhaps also when and why practice, conduct, thinking and self-image change. Determining when and why new beliefs are introduced provides a hint into the nous and psychological state of the physician whilst the rationale for their introduction and the physician’s self-appraisal of their impact on practice provides insights into their evolving value systems and priorities in the face of reflections, nous, insights, feedback and guided debriefs. This moves the discussion beyond concerns about mental health and burnout into the realm of holistic care of staff and patient safety.

Drawing on the notion that the costs of caring impact PIF and acknowledging the environmental, stakeholder and relational facets involved bring to the fore the importance of managing individual experiences, supporting physicians longitudinally and holistically, empowering them and building resilience. We have argued elsewhere on the merits of guided group^114^ and written reflections,^72^ longitudinal mentoring^115-117^ and use of portfolios^118^ to direct support to physicians in a timely and effective manner to contend with the notion of a changing sense of PIF.^119,120^ However, their use in these circumstances and in tandem with psychological support and regular debriefs,^70,73,114,121^ remains to be studied. Similarly, we believe creating a support network, ostensibly involving mentors and peer-mentors, should also be explored, given the evidence of their effectiveness in supporting mental well-being and PIF in other settings.

### Take Home Messages

• In summary, it is found in this study that physicians experience *costs of caring* that extend far beyond traditional notions of compassion fatigue but also include vicarious trauma, secondary traumatic stress that if left unaddressed, may chronically evolve to burnout.
• Regular, longitudinal and personalized support such as through mentorship is critical for physicians who contend with complex cases such as in the care of the death and dying.
• We propose the use of an RToP based model to understand the wider and far-reaching impact of the costs of caring on our personal conceptions as to what it means to be human.

### Limitations

Our study has several limitations. First, only English language papers were included, potentially limiting generalizability to non-Western regions where healthcare systems may differ. Second, although reviews were conducted by two independent research teams and overseen by senior physicians, there may be bias in article selection and analysis.

## Conclusion

The effects of the costs of caring are only just becoming clear. However, suggestions that they impact how palliative care physicians and oncologists view their professional and personal identities and thus their interactions with patients and their families and, in turn, patient safety and outcome underscore the need to better understand the costs of caring more holistically and longitudinally. A tool to evaluate these effects, the means to support physicians and preparation of their mentors must follow an effective and personalized study of physician experiences. This will be the focus of our coming work as we look forward to continued engagement in the practice space.

## Appendix 1

### Abbreviations

CF: Compassion Fatigue
VT: Vicarious Trauma
STS: Secondary Traumatic Stress
MD: Moral Distress
SSR: Systematic Scoping Review
SEBA: Systematic Evidence-Based Approach
PCC: Population, Context and Concept
MERSQI: Medical Education Research Study Quality Instrument
COREQ: Consolidated Criteria for Reporting Qualitative Research
PIF: Professional Identity Formation
